# On control of singleton attractors in multiple Boolean networks: integer programming-based method

**DOI:** 10.1186/1752-0509-8-S1-S7

**Published:** 2014-01-24

**Authors:** Yushan Qiu, Takeyuki Tamura, Wai-Ki Ching, Tatsuya Akutsu

**Affiliations:** 1 Advanced Modeling and Applied Computing Laboratory, Department of Mathematics, The University of Hong Kong, Pokfulam Road, Hong Kong; 2 Bioinformatics Center, Laboratory of Biological Information Networks, Bioinformatics Center, Institute for Chemical Research, Kyoto University, Uji, Kyoto 611-0011, Japan

## Abstract

**Background:**

Boolean network (BN) is a mathematical model for genetic network and control of genetic networks has become an important issue owing to their potential application in the field of drug discovery and treatment of intractable diseases. Early researches have focused primarily on the analysis of attractor control for a randomly generated BN. However, one may also consider how anti-cancer drugs act in both normal and cancer cells. Thus, the development of controls for multiple BNs is an important and interesting challenge.

**Results:**

In this article, we formulate three novel problems about attractor control for two BNs (i.e., normal cell and cancer cell). The first is about finding a control that can significantly damage cancer cells but has a limited damage to normal cells. The second is about finding a control for normal cells with a guaranteed damaging effect on cancer cells. Finally, we formulate a definition for finding a control for cancer cells with limited damaging effect on normal cells. We propose integer programming-based methods for solving these problems in a unified manner, and we conduct computational experiments to illustrate the efficiency and the effectiveness of our method for our multiple-BN control problems.

**Conclusions:**

We present three novel control problems for multiple BNs that are realistic control models for gene regulation networks and adopt an integer programming approach to address these problems. Experimental results indicate that our proposed method is useful and effective for moderate size BNs.

## Background

In the post-genome era, we observe rapid development in systems biology, a field focusing on interactions among the components of biological systems. *Gene regulatory networks *(*genetic networks*, in short) have been proposed to better understand the interaction of various kinds of genes, proteins and molecules. Many formalisms have been developed as models of genetic regulation processes, in particular, *Boolean network *(BN) has received substantial attention owing to its capacity to capture the switching behavior of genetic processes. Furthermore, its dynamic process is rich and complex and can provide insight into the global behavior of large genetic regulatory networks. A BN is a simple mathematical network: each gene (node) takes either 1 (active) or 0 (inactive), and the state of a gene is regulated by several genes called its input genes via its Boolean functions. It is to be noted that the states of genes can be updated synchronously and asynchronously. Thus *synchronous BNs *and *asynchronous BNs *have been proposed to model the two different behaviors. In this paper, synchronous BNs are considered since existing control studies of BNs are based on synchronous BNs, and detection of singleton attractor is equivalent for both models. Though synchronous BNs may be too simple compared with asynchronous BNs, they are effective to model and analyze some properties of real gene networks. Indeed, they have been used to analyze D. melanogaster embryo development, and the robustness of the genetic networks of *S. cerevisiae*, *E. coli*, *B. subtilis*, *D. melanogaster *and *A. thaliana *[1].

Many studies have been done on the analysis of *attractors *for randomly given BNs [2,3]. The main reason is that different attractors can be regarded as different cell types [2]. In [4-7], several approaches have been developed to efficiently identify and/or enumerate attractors in BNs. It was reported that detection of a singleton attractor (i.e., a fixed state) is an NP-hard problem [8]. Devloo et al. studied a method by transformation to a constraint satisfaction problem [4], while Irons proposed another method based on the use of small subnetworks [6]. Garg et al. [5] proposed a method based on Binary Decision Diagrams (BDDs). However, the average-case or worst-case complexity was not analyzed theoretically in these studies. Zhang et al. [7] developed algorithms to enumerate singleton and small attractors, and also studied the time complexities of average cases for these algorithms. Furthermore, Akutsu et al. [9] developed algorithms with guaranteed worst case time complexity for the singleton attractor detection for BNs with limited Boolean functions. In addition, Datta et al. [10,11] proposed methods for finding a control sequence for Probabilistic BNs (PBNs), a probabilistic extension of BNs. They defined the control problem with minimization of the sum of the total control cost and the cost of the final state. The cost of control is the cost to apply control inputs in some specified states, with the higher terminal costs corresponding to undesirable states. Their method is also applicable to finding actions of control for BNs, since BNs are special versions of PBNs. But, it is necessary for all these approaches to deal with 2*^n ^*× 2*^n ^*matrices, which makes it difficult to apply them to large-scale BNs. Consequently, Chen et al. [13] and Kobayashi and Hiraishi [14] proposed integer programming based approaches to variants of the PBN control problem. Akutsu et al. [15] also proposed an integer programming-based approach for constructing the attractor control problem for moderate size BNs. The *integer linear programming-based *(ILP) approach is effective to determine the optimal solutions subject to a series of constraints. Though [15] have shown that attractor control is ${\text{Σ}}_{2}^{p}\text{-hard}$, the ILP-based method can be applied to medium-size BNs. All of these works focused their approaches on a single randomly generated BN (i.e., one cell type), leaving the analysis of multiple BNs (various cell types) unaddressed. However, in real situations, there are different kinds of cell types, so it is more realistic to perform attractor analysis for multiple BNs. Thus, the development of attractor control problems for multiple BNs is an important and interesting challenge. Here, we propose an integer linear programming approach for constructing novel attractor controls for multiple BNs, and we provide three novel formulations of the attractor control problem to model various realistic cases. We consider simultaneous attractor control for multiple BNs and, specifically, focus on analyzing attractor control for two BNs that each corresponds to normal cells or cancer cells. Our objective, motivated by the fact that anti-cancer drugs act in both normal and cancer cells, is to find the same control (i.e., letting some genes be always active (1) and some genes always inactive (0)) for both networks. We aim at finding a control that can damage cancer cells significantly, while causing only limited damage to normal cells.

As another variant of attractor control, we find singleton attractors for normal cells with a guaranteed damaging level for cancer cells. In other words, we try to investigate whether there exists a control set that can damage cancer cells, while at the same time looking for a singleton attractor for normal cells under this control.

It is also of interest to consider finding a singleton attractor for cancer cells with limited damage to normal cells, to determine if there exists a control set ensuring no damage to normal cells, and under such a control set, to search for a singleton attractor for cancer cells.

To utilize ILP, all instances of the original problem are converted into ILP, to be able to apply an existing solver. These problems are transformed into ILP in a similar and systematic way to be shown later. The experimental results, using artificially generated BNs and realistic BNs, have demonstrated both the efficiency and the effectiveness of our proposed method.

## Problem formulations

In this section, we first give a brief introduction to BNs. Since the attractor control problem is based on the attractor detection problem (ATTRACTOR DETECTION), we begin with a brief introduction to that problem. We then define three variants of the attractor control problem: simultaneous attractor control for two BNs (cancer cell vs normal cell), attractor control for normal cells under the assumption of significant damaging cancer cells, attractor control for cancer cells under the assumption that normal cells are not damaged.

### Boolean networks

A Boolean Network (BN) *G*(*V*, *F *) is a set of vertices *V *= {*v*_1_, *v*_2_, ⋯, *v_n_*} and a list of Boolean functions *F *= {*f*_1_, *f*_2_, ⋯, *f_n_*} where *f_i _*: {0, 1}*^n ^*→ {0, 1}. Define *v_i_*(*t*) to be the state (0 or 1) of vertex *v_i _*at time *t*. The rules for the regulatory interactions among the genes are then expressed by

$$v_{i}\left(t+1\right)=f_{i}\left(v\left(t\right)\right),\;\;\;i=1,2,\;\cdots \;,\;n$$

where

$$v\left(t\right)=\left[v_{1}\left(t\right),\;\cdots \;,\;v_{n}\left(t\right)\right]$$

is called the *Gene Activity Profile *(GAP). *x *∧ *y*, *x *∨ *y*, *x *⊕ *y*, $\bar{x}$ is used to represent "AND" of *x *and *y*, "OR" of *x *and *y*, exclusive OR of *x *and *y*, and "NOT" of *x*, respectively. We denote *IN *(*v_i_*) as the set of relevant input nodes *v*_*i*1_, ⋯, *v_ik _*to *v_i_*. The number of relevant nodes incoming to *v_i _*is called as indegree of *v_i_*. *K *is used to represent the *maximum indegree *of a BN.

The following is an example of a BN.

$$\begin{array}{c} v_{1}\left(t+1\right)=v_{3}\left(t\right),\\ v_{2}\left(t+1\right)=\bar{v_{1}\left(t\right)},\\ v_{3}\left(t+1\right)=v_{1}\left(t\right)\wedge \bar{v_{2}\left(t\right)}. \end{array}$$

**Figure 1:**
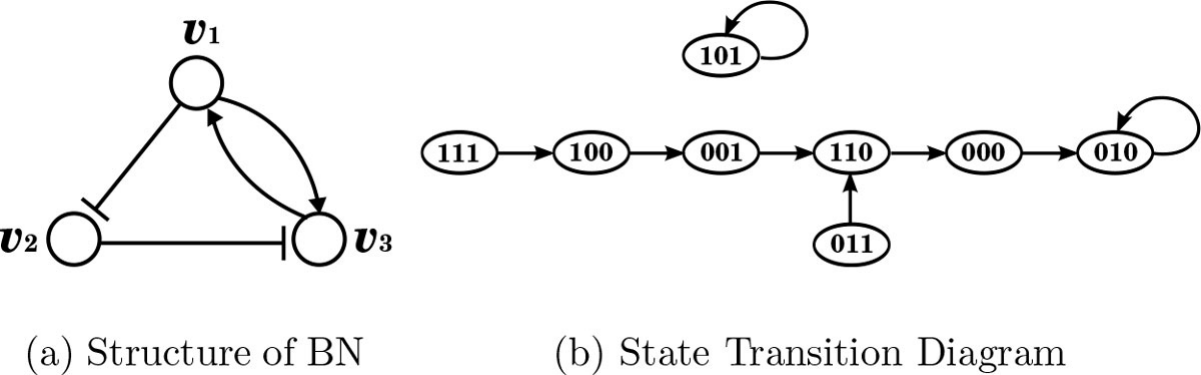
**Example of a Boolean network**.

Each gene *v_i _*is updated by a regulatory function *f_i_*. The corresponding truth table of a BN is given in Table 1. The dynamics of a BN and state transition diagram are shown in Figure 1. For example, the second row of the table means that if the state of BN is [0, 0, 1] at time *t*, then the state at time *t *+ 1 will be [1, 1, 0]. Similarly, the arc from 001 to 110 signifies that if the state of BN is [0, 0, 1] at time *t*, then the state will be [1, 1, 0] at time *t *+ 1. It is easily seen that a BN with *n *nodes has in total 2*^n ^*possible states. Thus, the state transition table and the state transition diagram have 2*^n ^*rows and 2*^n ^*vertices respectively.

**Table 1:** The truth table

State	*v*_1_(*t*)	*v*_2_(*t*)	*v*_3_(*t*)	*v*_1_(*t *+ 1)	*v*_2_(*t *+ 1)	*v*_3_(*t *+ 1)
1	0	0	0	0	1	0
2	0	0	1	1	1	0
3	0	1	0	0	1	0
4	0	1	1	1	1	0
5	1	0	0	0	0	1
6	1	0	1	1	0	1
7	1	1	0	0	0	0
8	1	1	1	1	0	0

**Table 2:** Results on Simultaneous Attractor Control

*n/m*	100/10	120/12	140/14	160/16
time(sec)	3.41	7.03	9.35	8.36
#pos/#rep	5/3.6	3/4.67	3/1	4/2
#neg/#rep	3/1	3/1	2/1	3/1

### Detection of an attractor

In a BN, the GAP **v**(*t *+ 1) at time *t *+ 1 is determined by the GAP **v**(*t*) at time *t*. When an initial GAP **v**(0) is given, a BN will finally converge to a set of global states (i.e., a directed cycle in the diagram of state transition). We call this set an *attractor*. If an attractor has only one state (i.e, **v **= **f(v)**), we call it *singleton attractor*, which is corresponding to a fixed point. Otherwise, if it consists of *p *distinct states, i.e.,

v(p)=f(f(⋯f(v(0))⋯)=v(0),

it is called a *cyclic attractor *of period *p*. We can see from Figure 1 that 010 and 101 are two singleton attractors.

Here, the problem of attractor detection is defined to find an attractor for a given BN. It should be noted that finding attractors with large periods is not easy. Thus we study attractor detection in which upper bound of the period is limited by some given threshold *pmax*. We define this problem as follows. Here we consider the case of *p_max _*= 1 (i.e., the *singleton attractor detection problem*).

**Definition 1**: [ATTRACTOR DETECTION]

Instance: a BN and *p_max_*, the maximum period.

Problem: Output an attractor whose period is at most *p_max_*. If such an attractor does not exist, "NULL" should be the output.

For a BN of Table 1, ATTRACTOR DETECTION should output either 010 or 101. Since we only consider the case of *p_max _*= 1 hereafter, we also use *v_i _*to denote the (steady) state of *v_i_*.

### Simultaneous attractor control for multiple Boolean networks

A control problem for a single BN was proposed by Akutsu et al. [15]. We consider whether there exists a control for multiple BNs. We consider two BNs, *N*_1_(*V*, *F*_1_) and *N*_2_(*V*, *F*_2_), representing a cancer cell and a normal cell, respectively. Here, *V *is a set of genes (nodes) while *F*_1 _and *F*_2 _are sets of regulation functions (i.e., sets of Boolean functions along with input genes) for *N*_1 _and *N*_2_, respectively. We try to find the same control (i.e., some genes always active (1) and some genes always inactive (0)) for both networks, considering that anti-cancer drugs act in both normal and cancer cells. Therefore, we set our endpoint as finding a control that causes limited damage to normal cells but significant damage to cancer cells. Suppose control nodes are chosen as $v_{i_{1}},\;\cdots \;,\;v_{i_{m}}$ and these nodes are assigned by Boolean values of $b_{i_{1}},\;\cdots \;,\;b_{i_{m}}$. Then, we call **v **as a singleton attractor if it satisfies the following conditions (denoted by COND1):

(i) *v_i _*= *b_i_*; if *i *∈ {*i*_1_, ⋯, *i_m_*},

(ii) *v_i _*= *f_i_*(**v**); otherwise.

Furthermore, in order to evaluate how appropriate each attractor state is, we need a score function *h*(**v**) for cancer cells and *g*(**v**) for normal cells from {0, 1}*^n ^*to the set of real numbers. For simplicity, we assume these functions are given in linear form as follows:

$$h\left(v\right)=\underset{i}{\sum }\alpha_{i}\;\cdot \;\left(1-w_{i}\right)\;\cdot \;v_{i}\;\;\;\text{and}\;\;\;g\left(v\right)=\underset{i}{\sum }\beta_{i}\;\cdot \;\left(1-w_{i}\right)\;\cdot \;v_{i}$$

where *w_i _*= 1 (resp., *w_i _*= 0) holds if *v_i _*is selected (resp., not selected) as a control node. This means that we do not take the scores of the selected control nodes into account for the score functions (*h*(**v**) and *g*(**v**)). Here, *α_i _*and *β_i _*are real constants. For example, if *α_i _*= 1 for all *i*, then **v**(*t*) = (1, 1, ⋯, 1) is the state with the highest score (i.e., the desired state for the cancer cells). Similarly, if *β_i _*= -1 for all *i*, then **v**(*t*) = (0, 0, ⋯, 0) is the state with the highest score (i.e., the desired state for the normal cells). We define

G(v)=h(v)+g(v)

as the final score function for these two networks. Since the singleton attractors may not be uniquely determined, considering the worst case, the minimum score of singleton attractors is necessary to be maximized. Because it is difficult to give a direct formulation of this problem, the problem of simultaneous control of attractors is defined with *θ*, which is a threshold of the minimum score, as follows.

**Definition 2 **: [SIMULTANEOUS ATTRACTOR CONTROL] (SAC)

Instance: 2 BNs, the number of control nodes *m*, a score function *G*, and a threshold *θ*.

Problem: Find *m *control nodes and 0-1 assignment for them where the minimum score of singleton attractors is greater than *θ*. If no such nodes exist, then "NULL" is the output.

We give an example about attractor control for a given BN, say, the BN described in Table 1. We assume *α*_1 _= 1, *α*_2 _= 2, *α*_3 _= 3 and *m *= 1. If *v*_1 _is a control node and 1 is assigned to this node, there exists one singleton attractor 101 with score 3. If 0 is assigned to *v*_2_, two singleton attractors 000 and 101 exist. Note that the scores of 000 and 101 are 0 and 4, respectively, so the minimum score is 0. Then, if 0 is assigned to *v*_3_, 010 is a singleton attractor with score 2. Though checking the other three cases is necessary, we can conclude that the first case (assigning 1 to *v*_1_) gives the solution.

### Attractor control for normal cells under the assumption of significant damage to cancer cells

We consider another attractor control variant for multiple networks. We try to investigate whether there exists a control that can guarantee significant damage to cancer cells (*N*_1_) and a singleton attractor for normal cells (*N*_2_). To ensure that the control can cause significant damage to the cancer cells, we introduce a threshold *ξ*_1 _for *N*_1 _to be given later. The score function *G*(**v**) becomes *g*(**v**). It is possible that there exist multiple singleton attractors for *N*_2_, so we maximize the minimum score of the singleton attractors and give the threshold *θ*_1 _for the minimum score. The problem is formulated as follows:

**Definition 3: **[Attractor Control under Damaging Cancer cells substantially] (ACDC)

Instance: 2 BNs, a score function *G*, the number of control node *m*, and thresholds *θ*_1 _and *ξ*_1_,

Problem: Find 0-1 assignment to *m *control nodes where the minimum score of singleton attractors of *N*_2 _is no less than *θ*_1_, and the score of the singleton attractor for *N*_1 _is greater than *ξ*_1_, respectively. If such nodes do not exist, then "NULL" is the output.

### Attractor control for cancer cells under the assumption of limited damage to normal cells

We also investigate whether there exists a control that ensures limited damage to the normal cells (*N*_2_), and whether there still exists a singleton attractor for cancer cells (*N*_1_). We give a threshold *ξ*_2 _for the normal cells that guarantees keeping the normal cells undamaged and convert the score function into *G*(**v**)=*h*(**v**). In order to obtain the unique singleton attractor for *N*_1_, we consider maximizing the minimum score function integrating the threshold *θ*_2 _for the minimum score of *N*_1_.

**Definition 4: **[Attractor Control under Keeping Normal cells undamaged] (ACKN)

Instance: 2 BNs, a score function *G*, the number of control node *m*, and thresholds *θ*_2 _and *ξ*_2_,

Problem: Find *m *nodes and a 0-1 assignment of these control nodes for which the minimum score of singleton attractors for *N*_1 _is no less than *θ*_2 _and the score of singleton attractors for *N*_2 _is greater than *ξ*_2_, respectively. If such nodes do not exist, then "NULL" is the output.

## Methods

Integer programming, in particular, *integer linear programming *(ILP) is to maximize (or minimize) a linear objective function with linear constraints (i.e., linear inequalities and linear equations) with all the variables taking integer values. From here on, either 0 or 1 is assigned to each variable, representing the Boolean values. Furthermore, we introduce *x_i _*and *si *to represent a 0-1 variable that corresponds to *v_i _*for *N*_1 _and *N*_2_, respectively.

### ILP for ATTRACTOR DETECTION

Here we review how ATTRACTOR DETECTION is formalized as ILP in [15]. Define *δ_b_*(*x*) by

$$\delta_{b}\left(x\right)=\left\{\begin{array}{c} x\;\;\text{if}\;b=1\\ \bar{x}\;\;\text{otherwise}. \end{array}\right)$$

Then if Boolean function has *k *inputs, we represent it by

$$f_{i}\left(x_{i_{1}},\;\cdots \;,\;x_{i_{k}}\right)=\underset{b_{i_{1}}\cdots b_{i_{k}}\in {\left\{0,1\right\}}^{k}}{\vee }f_{i}\left(b_{i_{1}},\;\cdots \;,\;b_{i_{k}}\right)\wedge \delta_{b_{1}}\left(x_{i_{1}}\right)\wedge \cdots \wedge \delta_{b_{k}}\left(x_{i_{k}}\right).$$

Since Boolean constraints cannot be used in ILP directly, we define *τ_b_*(*x*) as

$$\tau_{b}\left(x\right)=\left\{\begin{array}{cc} x & \text{if}\;b=1\\ 1-x & \text{otherwise}. \end{array}\right)$$

We introduce $x_{i,b_{i_{1}}\cdots b_{i_{k}}}$ in order to represent whether each 0-1 assignment for $\left(b_{i_{1}},\;\cdots \;,\;b_{i_{k}}\right)$ with $f_{i}\left(b_{i_{1}},\;\cdots \;,\;b_{i_{k}}\right)=1$ is satisfied. If $f_{i}\left(b_{i_{1}},\;\cdots \;,\;b_{i_{k}}\right)=1$, we give constraints

$$\begin{array}{c} x_{i,b_{i_{1}}\cdots b_{i_{k}}}\geq \left(\sum_{j=1,\cdots \;,k}\tau_{b_{i_{j}}}\left(x_{i_{j}}\right)\right)-\left(k-1\right),\\ x_{i,b_{i_{1}}\cdots b_{i_{k}}}\leq \frac{1}{k}\sum_{j=1,\cdots \;,k}\tau_{b_{i_{j}}}\left(x_{i_{j}}\right), \end{array}$$

in which the former constraint forces $x_{i,b_{i_{1}}\cdots b_{i_{k}}}$ to be 1 if $\delta_{b_{1}}\left(x_{i_{1}}\right)\wedge \cdots \wedge \delta_{b_{k}}\left(x_{i_{k}}\right)$ is satisfied, and the latter forces $x_{i,b_{i_{1}}\cdots b_{i_{k}}}$ to be 0 if it is not satisfied. If $f_{i}\left(b_{i_{1}},\;\cdots \;,\;b_{i_{k}}\right)=0$, a constraint $x_{i,b_{i_{1}}\cdots b_{i_{k}}}=0$ is simply added. These constraints guarantee that

$$x_{i,b_{i_{1}}\cdots b_{i_{k}}}=1$$

if and only if

$$f_{i}\left(b_{i_{1}},\;\cdots \;,\;b_{i_{k}}\right)\wedge \delta_{b_{1}}\left(x_{i_{1}}\right)\wedge \cdots \wedge \delta_{b_{k}}\left(x_{i_{k}}\right)=1.$$

Finally, for every *x_i_*, we add the following constraints

$$x_{i}\leq \underset{b_{i_{1}}\cdots b_{i_{k}}\in {\left\{0,1\right\}}^{k}}{\sum }x_{i,b_{i_{1}}\cdots b_{i_{k}}}$$

and

$$x_{i}\geq \frac{1}{2^{k}}\underset{b_{i_{1}}\cdots b_{i_{k}}\in {\left\{0,1\right\}}^{k}}{\sum }x_{i,b_{i_{1}}\cdots b_{i_{k}}}.$$

The above constraints are included so as to ensure that $x_{i}=f\left(x_{i_{1}},\;\cdots \;,\;x_{i_{k}}\right)$ holds for each *x_i_*. Thus any feasible solution corresponds to a singleton attractor. A simple example is given to illustrate this method. Assume *x*_1 _is determined by $x_{1}=f_{1}\left(x_{2},\;x_{3}\right)=x_{2}\vee {\bar{x}}_{3}$. Then *f*_1_(*x*_2_, *x*_3_) can be represented as

$$\begin{array}{cc} f_{1}\left(x_{2},\;x_{3}\right) & =\;\left(f_{1}\left(0,0\right)\wedge {\bar{x}}_{2}\wedge {\bar{x}}_{3}\right)\vee \left(f_{1}\left(0,1\right)\wedge {\bar{x}}_{2}\wedge x_{3}\right)\\ & \;\left(f_{1}\left(1,0\right)\wedge x_{2}\wedge {\bar{x}}_{3}\right)\vee \left(f_{1}\left(1,1\right)\wedge x_{2}\wedge x_{3}\right)\\ & =\;\left({\bar{x}}_{2}\wedge {\bar{x}}_{3}\right)\vee \left(x_{2}\wedge {\bar{x}}_{3}\right)\vee \left(x_{2}\wedge x_{3}\right). \end{array}$$

Thus, this Boolean function can be converted into the following inequalities:

$$\left\{\begin{array}{ccc} x_{1,00} & \geq & \left(1-x_{2}\right)+\left(1-x_{3}\right)\;-1=-x_{2}-x_{3}+1,\\ x_{1,00} & \leq & \frac{1}{2}\left(1-x_{2}+1-x_{3}\right),\\ x_{1,01} & = & 0,\\ x_{1,10} & \geq & x_{2}+\left(1-x_{3}\right)\;-1=x_{2}-x_{3},\\ x_{1,10} & \leq & \frac{1}{2}\left(x_{2}+1-x_{3}\right),\\ x_{1,11} & \geq & x_{2}+x_{3}-1,\\ x_{1,11} & \leq & \frac{1}{2}\left(x_{2}+x_{3}\right),\\ x_{1} & \leq & x_{1,00}+x_{1,01}+x_{1,10}+x_{1,11},\\ x_{1} & \geq & \frac{1}{4}\left(x_{1,00}+x_{1,01}+x_{1,10}+x_{1,11}\right). \end{array}\right)$$

It is easily seen from this example that the transformation is correct. Integrating all the constraints, we can formulate the integer programming for singleton attractor detection as below.

***maximize *** *x*_2_

subject to

$$\left\{\begin{array}{c} x_{i,b_{i_{1}}\cdots b_{i_{k}}}\geq \left(\sum_{j=1,\cdots \;,k}\tau_{b_{i_{j}}}\left(x_{i_{j}}\right)\right)-\left(k-1\right),\\ x_{i,b_{i_{1}}\cdots b_{i_{k}}}\leq \frac{1}{k}\sum_{j=1,\cdots \;,k}\tau_{b_{i_{j}}}\left(x_{i_{j}}\right),\\ \;\text{for}\;\text{all}\;i\in \left[1\cdots n\right]\;\text{and}\;b_{i_{1}}\cdots b_{i_{k}}\in {\left\{0,1\right\}}^{k},\text{such}\;\text{that}\;f_{i}\left(b_{i_{1}},\;\cdots \;,\;b_{i_{k}}\right)=1,\\ x_{i,b_{i_{1}}\cdots b_{i_{k}}}=0,\;\text{for}\;\text{all}\;i\in \left[1\cdots n\right]\;\text{and}\;b_{i_{1}}\cdots b_{i_{k}}\in {\left\{0,1\right\}}^{k},\;\text{such}\;\text{that}\;f_{i}(b_{i_{1}},\;\cdots \;,\;b_{i_{\text{k}}}\text{)}\;\text{ = }\;\text{0,}\\ x_{i}\leq \sum_{b_{i_{1}},\cdots b_{i_{k}}\in {\left\{0,1\right\}}^{k}}x_{i,b_{i_{1}}\cdots b_{i_{k}}},\;\text{for}\;\text{all}\;i\in \left[1\cdots n\right],\\ x_{i}\geq \frac{1}{2^{k}}\sum_{b_{i_{1}},\cdots b_{i_{k}}\in {\left\{0,1\right\}}^{k}}x_{i,b_{i_{1}}\cdots b_{i_{k}}},\\ x_{i}\in \left\{0,1\right\},\;\text{for}\;\text{all}\;i\in \left[1\cdots n\right]\\ x_{i,b_{i_{1}}\cdots b_{i_{k}}}\in \left\{0,1\right\},\;\text{for}\;\text{all}\;i\in \left[1\cdots n\right]\;\text{and}\;b_{i_{1}}\cdots b_{i_{k}}\in {\left\{0,1\right\}}^{k}. \end{array}\right)$$

We note that ATTRACTOR DETECTION is a decision problem, not an optimization problem, so we do not need an objective function, but we will define an objective function in order to apply ILP. Here, we give the objective function simply as "Maximize *x*_2_". We can extend the above method to detect a cyclic attractor whose period is at most *p_max_*, but for that purpose, we will define many more variables $x_{i,t},\;x_{i,t,b_{i_{1}},\cdots \;,b_{i_{k}}}\text{for}\;t\in \left\{0,1,\;\cdots \;,p_{max}-1\right\}$.

### ILP for SIMULTANEOUS ATTRACTOR CONTROL

Consider that each variable *vi *has two possibilities, i.e,

(i) *v_i _*is not chosen for a control node (i.e., *v_i _*corresponds to an internal node),

(ii) *v_i _*is chosen for a control node (i.e., *v_i _*becomes an external node).

In order to specify the state of a variable, we introduce the following variables and constraints for *N*_1_.

Let *x_i _*be

$$x_{i}=\left\{\begin{array}{c} p_{i}\;\;\;\text{if}\;\;\;w_{i}=0;\\ z_{i}\;\;\;\text{if}\;\;\;w_{i}=1. \end{array}\right)$$

This function can be represented by

$$\begin{array}{c} p_{i}-w_{i}\leq x_{i}\leq p_{i}+w_{i},\\ z_{i}-\left(1-w_{i}\right)\leq x_{i}\leq z_{i}+\left(1-w_{i}\right). \end{array}$$

The above representation means that *w_i _*= 1 corresponds to the case that *x_i _*is selected as a control node, to which *z_i _*gives a 0-1 assignment. Similarly, for *N*_2 _of the normal cell, another variable *s_i _*is defined as

$$s_{i}=\left\{\begin{array}{c} q_{i}\;\;\;\;\text{if}\;\;\;\;w_{i}=0;\\ z_{i}\;\;\;\;\text{if}\;\;\;\;w_{i}=1. \end{array}\right)$$

This function can also be represented by

$$\begin{array}{c} q_{i}-w_{i}\leq s_{i}\leq q_{i}+w_{i}\\ z_{i}-\left(1-w_{i}\right)\leq s_{i}\leq z_{i}+\left(1-w_{i}\right). \end{array}$$

In this representation, we can see that *w_i _*= 1 also corresponds to the case that *si *is selected as a control node, and has the same 0-1 assignment. This guarantees that these two different BNs are under the same control. For this attractor control problem, we assume that the score functions for *N*_1 _and *N*_2 _take the following forms, respectively:

$$h\left(x\right)=\underset{i}{\sum }\alpha_{i}\;\cdot \;\left(1-w_{i}\right)\;\cdot \;x_{i}$$

and

$$g\left(s\right)=\underset{i}{\sum }\beta_{i}\;\cdot \;\left(1-w_{i}\right)\;\cdot \;s_{i}.$$

We can assume without loss of generality that *α_i _*≥ 0 and *β_i _*≤ 0. Then, for the first BN, *N*_1_, we try to maximize the score of the internal nodes, so we need to maximize *h*(*x*). The score function can be reduced to ${\text{Σ}}_{i}\;\alpha_{i}\;\cdot \;u_{i}$ if we add the constraints *u_i _*≤ *x_i _*and *u_i _*≤ 1 - *w_i_*, where *u_i _*is a binary variable. In terms of maximizing the score function *g*(*s*) for *N*_2_, we introduce the additional binary variable *γ_i _*such that *γ_i _*= -*β_i_*. Thus, the score function for *g*(*s*) becomes ${\text{Σ}}_{i}\gamma_{i}\;\cdot \;\left(1-w_{i}\right)\;\cdot \;\left(1-s_{i}\right)$. Furthermore, the score function can be converted into ${\text{Σ}}_{i}\;\gamma_{i}\;\cdot \;r_{i}$, if we add the constraints *r_i _*≤ (1 - *w_i_*) and *r_i _*≤ (1 - *s_i_*).

The original aim is that the minimum score of singleton attractors is maximized for these two networks. However, it is difficult to give a direct ILP formalization for this problem, because of the ${\text{Σ}}_{2}^{p}\text{-hardness}$ of the problem, so as in [15], we firstly formalize an ILP to find a singleton attractor with the maximum score by choosing and controlling *m *nodes. Incorporating the inequalities mentioned above with the ILP formalization for ATTRACTOR DETECTION, the following ILP formalization for SAC is obtained.

**maximize**$\sum_{i}\alpha_{i}\;\cdot \;u_{i}+\gamma_{i}\;\cdot \;r_{i}$

*subject to*

$$\left\{\begin{array}{c} x_{i,b_{i_{1}}\cdots b_{i_{k}}}\geq \left(\sum_{j=1,\cdots \;,k}\tau_{b_{i_{j}}}\left(x_{i_{j}}\right)\right)-\left(k-1\right),\\ x_{i,b_{i_{1}}\cdots b_{i_{k}}}\leq \frac{1}{k}\sum_{j=1,\cdots \;,k}\tau_{b_{i_{j}}}\left(x_{i_{j}}\right),\\ \;\text{for}\;\text{all}\;i\in \left[1\cdots n\right]\;\text{and}\;b_{i_{1}}\cdots b_{i_{k}}\in {\left\{0,1\right\}}^{k}\text{such}\;\text{that}\;f_{i}\left(b_{i_{1}},\;\cdots \;,\;b_{i_{k}}\right)=1\\ x_{i,b_{i_{1}}\cdots b_{i_{k}}}=0,\\ \;\text{for}\;\text{all}\;i\in \left[1\cdots n\right]\;\text{and}\;b_{i_{1}}\cdots b_{i_{k}}\in {\left\{0,1\right\}}^{k}\;\text{such}\;\text{that}\;f_{i}\left(b_{i_{1}},\;\cdots \;,\;b_{i_{k}}\right)=0\\ p_{i}\leq \sum_{b_{i_{1}},\cdots b_{i_{k}}\in {\left\{0,1\right\}}^{k}}x_{i,b_{i_{1}}\cdots b_{i_{k}}},\;\text{for}\;\text{all}\;i\in \left[1\cdots n\right]\\ p_{i}\geq \frac{1}{2^{k}}\sum_{b_{i_{1}},\cdots b_{i_{k}}\in {\left\{0,1\right\}}^{k}}x_{i,b_{i_{1}}\cdots b_{i_{k}}},\\ p_{i}-w_{i}\leq x_{i}\leq p_{i}+w_{i},\\ z_{i}+w_{i}-1\leq x_{i}\leq z_{i}-w_{i}+1,\\ u_{i}\leq x_{i},\;u_{i}\leq 1-w_{i},\;\text{for}\;\text{each}\;i\in \left[1\cdots n\right]\\ s_{i,b_{i_{1}}\cdots b_{i_{k}}}\geq \left(\sum_{j=1,\cdots \;,k}\tau_{b_{i_{j}}}\left(s_{i_{j}}\right)\right)-\left(k-1\right),\\ s_{i,b_{i_{1}}\cdots b_{i_{k}}}\leq \frac{1}{k}\left(\sum_{j=1,\cdots \;,k}\tau_{b_{i_{j}}}\left(s_{i_{j}}\right)\right),\\ \;\text{for}\;\text{all}\;i\in \left[1\cdots n\right]\;\text{and}\;b_{i_{1}}\cdots b_{i_{k}}\in {\left\{0,1\right\}}^{k}\text{such}\;\text{that}\;f_{i}\left(b_{i_{1}},\;\cdots \;,\;b_{i_{k}}\right)=1\\ s_{i,b_{i_{1}}\cdots b_{i_{k}}}=0,\;\text{for}\;\text{all}\;i\in \left[1\cdots n\right]\;\text{and}\;b_{i_{1}}\cdots b_{i_{k}}\in {\left\{0,1\right\}}^{k}\;\text{such}\;\text{that}\;f_{i}\left(b_{i_{1}},\;\cdots \;,\;b_{i_{k}}\right)=0\\ q_{i}\leq \sum_{b_{i_{1}},\cdots b_{i_{k}}\in {\left\{0,1\right\}}^{k}}s_{i,b_{i_{1}}\cdots b_{i_{k}}},\;\text{for}\;\text{all}\;i\in \left[1\cdots n\right]\\ q_{i}\geq \frac{1}{2^{k}}\sum_{b_{i_{1}},\cdots b_{i_{k}}\in {\left\{0,1\right\}}^{k}}s_{i,b_{i_{1}}\cdots b_{i_{k}}},\\ q_{i}-w_{i}\leq s_{i}\leq q_{i}+w_{i},\\ z_{i}+w_{i}-1\leq s_{i}\leq z_{i}-w_{i}+1,\\ r_{i}\leq 1-s_{i},\;r_{i}\leq 1-w_{i},\;\text{for}\;\text{each}\;i\in \left[1\cdots n\right]\\ x_{i},\;s_{i},p_{i},\;q_{i},\;z_{i},\;w_{i},\;u_{i},\;r_{i}\in \left\{0,1\right\},\;\text{for}\;\text{all}\;i\in \left[1\cdots n\right],\\ x_{i,b_{i_{1}}\cdots b_{i_{k}}}\in \left\{0,1\right\},\;\text{for}\;\text{all}\;i\in \left[1\cdots n\right]\;\text{and}\;b_{i_{1}}\cdots b_{i_{k}}\in {\left\{0,1\right\}}^{k}\\ s_{i,b_{i_{1}}\cdots b_{i_{k}}}\in \left\{0,1\right\},\;\text{for}\;\text{all}\;i\in \left[1\cdots n\right]\;\text{and}\;b_{i_{1}}\cdots b_{i_{k}}\in {\left\{0,1\right\}}^{k}\\ \sum_{i=1,\cdots \;,n}w_{i}=m. \end{array}\right)$$

We denote this ILP formulation as ILP-A. Since singleton attractors are not always uniquely determined, considering the worst case, it is necessary to maximize the minimum score of the singleton attractors. Suppose that $V^{\prime }=\left(v_{i_{1}},\;\cdots \;,\;v_{i_{m}}\right)$ has been selected as the control nodes with 0-1 value $B^{\prime }=\left(b_{i_{1}},\;\cdots \;,\;b_{i_{m}}\right)$. These notions are also used in the following two problems, so detection of the attractor with the minimum score with these control nodes can be formulated as follows. Define *I *= {*i*_1_, ⋯, *i_m_*}. The objective function can be replaced by

$$\text{“Minimize }\underset{i\notin I}{\sum }\alpha_{i}x_{i}+\gamma_{i}\left(1-s_{i}\right)”$$

and the constraints replaced *u_i _*and *r_i _*by

$$\left\{\begin{array}{cc} x_{i}=z_{i},\;s_{i}=z_{i},\;w_{i}=1,\;z_{i}=b_{i} & \text{for}\;\text{all}\;\;\;\;i\in I\\ w_{i}=0 & \text{for}\;\text{all}\;\;\;\;i\notin I. \end{array}\right)$$

The resulting ILP is denoted by ILP-A'.

From the perspective of avoiding examination of the examined (*V*′, *B*′), we will modify ILP-A. In other words, we try to find node-value pairs that are not the same as the previously obtained solutions. This can be tackled by some additional linear inequalities which ensures that the following node-value pairs differ from the previous ones. Note that these two networks have the same control, so if we can avoid obtaining the previously examined (*V*′, *B*′) for *N*_1_, then we can also get different node-value pairs for *N*_2_. Thus, we shall consider one of the networks, i.e., *N*_1_. Assume that $x^{k}=\left(x_{1}^{\left(k\right)},\;x_{2}^{\left(k\right)},\;\cdots \;,\;x_{n}^{\left(k\right)}\right)$ is the *k*th control previously found, where we let $x_{i}^{\left(k\right)}=z_{i}^{\left(k\right)}\;\text{if}\;w_{i}=1$, and otherwise $x_{i}^{\left(k\right)}=-1$. Then for each *k*, we add the following linear inequality:

$$\underset{x_{i}^{\left(k\right)}\neq -1}{\sum }\left(\delta \left(x_{i}^{\left(k\right)},\;1\right)\left(-z_{i}^{\left(k+1\right)}-w_{i}^{\left(k+1\right)}\right)+\delta \left(x_{i}^{\left(k\right)},\;0\right)\left(z_{i}^{\left(k+1\right)}-w_{i}^{\left(k+1\right)}\right)\right)\geq 1-\underset{x_{i}^{\left(k\right)}\neq -1}{\sum }\left(1+x_{i}^{\left(k\right)}\right),$$

where *δ*(*x*, *y*) is the delta function,

$$\delta \left(x,y\right)=\left\{\begin{array}{ccc} 1 & \;\text{if}\; & x=y\\ 0 & \;\text{if} & x\neq y. \end{array}\right)$$

This inequality guarantees that the following must hold for at least one of the control nodes:

• if $x_{i}^{\left(k\right)}=1$, either $z_{i}^{\left(k+1\right)}=0$ or $w_{i}^{\left(k+1\right)}=0$ holds,

• otherwise, either $z_{i}^{\left(k+1\right)}=1$ or $w_{i}^{\left(k+1\right)}=0$ holds.

We consider the case that one of the control nodes $x_{i}^{\left(k\right)}$ is $\left(\text{i}.\text{e}.,\;z_{i}^{\left(k\right)}=1\right)$ for the *k*th control. If $w_{i}^{\left(k+1\right)}=0$ holds for the (*k *+ 1)th control, then in the next iteration, it will not be selected as a control node. Otherwise, $w_{i}^{\left(k+1\right)}=1$, so it is still a control node. However, the inequality guarantees that $z_{i}^{\left(k+1\right)}$ must be 0, which is different from the previous value $\left(z_{i}^{\left(k\right)}=1\right)$. Thus, the above inequality ensures that the following obtained set of node-value pairs is different from the previous ones. Define ILP-B as this modified version in the following context. We can solve the simultaneous attractor control problem for multiple networks through the following algorithm.

1. Repeat steps 2 - 3.

2. Find (*V'*, *B'*) yielding the maximum score of a singleton attractor using ILP-B where (*V'*, *B'*) is not the same as any of the already examined nodes/values pairs. "NULL" should be output and halt, if the maximum score is less than *θ*.

3. For (*V'*, *B'*), calculate the singleton attractors with the minimum score by ILP-A'. Output (*V'*, *B'*) and halt, if the minimum score is no less than *θ*.

Note that in the worst case, it may repeat this procedure exponentially many times, but we expect that the procedure will not be repeated so many times, since the expected number of singleton attractors (i.e, per (*V'*, *B'*)) is small, regardless of the total number of nodes (*n*). How to select the thresholds *θ *is an important issue in this program. If we know an appropriate threshold in advance, we can simply use such a *θ*. Here, we let *θ *be 1.2*n*, because the desired attractors almost always exist if *θ *≪ 1.2*n*, and it often occurs in our preliminary computational experiments that the algorithm cannot find the desired attractors if *θ *≫ 1.2*n*.

### ILP for attractor control under damaging cancer cells substantially

We try to investigate if there exists a control that ensures damaging the cancer cell substantially and, under this condition, identifying singleton attractors for the normal cell. For the ILP-A of ACDC (Attractor Control under Damaging Cancer cells substantially), we have to make sure that the control damages the cancer cell significantly by introducing the following inequality

(1) $$\underset{i}{\sum }\gamma_{i}u_{i}\geq \xi_{1}.$$

A larger *ξ*_1 _signifies that the cancer cell is damaged substantially. Here we set the *ξ*_1 _as 0.6*n*. Then, for the normal cell, we define the objective function as $\sum_{i}\gamma_{i}\cdot \;r_{i}$. It should be noted that for this problem it is possible that there does not exist any singleton attractor for *N*_2 _since the control set must satisfy the inequality (1) ensuring significant damage to the cancer cells. In terms of ILP-A', we aim at finding the maximum of the minimum score of *N*_2 _for the case of having multiple singleton attractors. Since we consider the multiple singleton attractors for *N*_2_, we do not need to include constraints about *N*_1_, so we can replace *r_i _*with

$$\begin{array}{ccc} s_{i}=z_{i},\;w_{i}=1,\;z_{i}=b_{i} & \;\text{for}\;\text{all} & i\in I\\ w_{i}=0 & \text{for all} & i\notin I \end{array}$$

Consequently, the objective function becomes "Minimize $\sum_{i\notin I}\gamma i\left(\text{1}-s_{i}\right)$". As for ILP-B, to avoid examining the previously obtained (*V'*, *B'*) for *N*_2_, we assume that $s^{k}=\left(s_{1}^{\left(k\right)},s_{2}^{\left(k\right)},\ldots ,s_{n}^{\left(k\right)}\right)$ is the *k*th control previously found, where we let $s_{i}^{\left(k\right)}=s_{i}^{\left(k\right)}$ if *w_i _*= 1, otherwise $s_{i}^{\left(k\right)}=-1$. Then for each *k*, we add the

following linear inequality:

$$\underset{s_{i}^{\left(k\right)}\neq -1}{\sum }\left(\delta \left(s_{i}^{\left(k\right)},1\right)\left(-z_{i}^{\left(k+1\right)}-w_{i}^{\left(k+1\right)}\right)+\delta \left(s_{i}^{\left(k\right)},0\right)\left(z_{i}^{\left(k+1\right)}-w_{i}^{\left(k+1\right)}\right)\right)\geq 1-\underset{s_{i}^{\left(k\right)}\neq -1}{\sum }\left(1+s_{i}^{\left(k\right)}\right),$$

We can solve this problem using the following algorithm.

1. Repeat steps 2 - 3.

2. Find (*V'*, *B'*) yielding the maximum score of a singleton attractor using ILP-B where (*V'*, *B'*) is not the same as any of the already examined nodes/values pairs and that the control damages the cancer cell significantly. "NULL" should be output and halt, if the singleton attractor does not exist.

3. For (*V'*, *B'*), calculate the singleton attractors with the minimum score by ILP-A'. Output (*V'*, *B'*) and halt, if the minimum score is greater than *θ*_1_.

Here, we set *θ*_1 _to be 0.6*n*, because if *θ*_1 _≪ 0.6*n*, then the desired attractor almost always exists, whereas if *θ*_1 _≫ 0.6*n*, then the desired attractor seldom exists.

### ILP for attractor control under keeping normal cells undamaged

We also try to investigate whether there exists a control that ensures not damaging the normal cell substantially and under this condition, we find a singleton attractor for the cancer cell. In terms of ILP-A for ACKN (Attractor Control under Keeping Normal cells undamaged), considering this control should not damage the normal cell substantially, so we introduce an additional inequality

(2) $$\underset{i}{\sum }\gamma_{i}r_{i}\geq \;\xi_{\text{2}}.$$

We set the *ξ*_2 _to be 0.6*n*, and we define the objective function as $\sum_{i}\alpha_{i}\;\cdot \;u_{i}$. The singleton attractor for *N*_1 _is difficult to find for this problem, because the control set must satisfy the inequality (2) (not damaging the normal cell substantially). Considering that there may exist multiple singleton attractors for the cancer cell, and the worst case, it is necessary to maximize the minimum score of singleton attractors.

Thus for ILP-A', we do not need to add any constraints about *N*_2 _for ILP-A' so we replace *u_i _*by

$$\begin{array}{ccc} x_{i}=z_{i},\;w_{i}=1,\;z_{i}=b_{i} & \;\text{for}\;\text{all} & i\in I\\ w_{i}=0 & \text{for all} & i\notin I \end{array}$$

The objective function is "Minimize $\sum_{i\notin I}\alpha_{i}x_{i}.$ Except these modifications, ILP-B is exactly the same as in the case of SAC. We can solve this problem through the following algorithm.

1. Repeat steps 2 - 3.

2. Find (*V'*, *B'*) yielding the maximum score of a singleton attractor using ILP-B where (*V'*, *B'*) is not the same as any of the already examined nodes/values pairs and that the control causes only limited damage to the normal cell. "NULL" should be output and halt, if no singleton attractor for *N*_1 _exist.

3. For (*V'*, *B'*), calculate the singleton attractors with the minimum score by ILP-A'. Output ((*V'*, *B'*) and halt, if the minimum score is not less than *θ*_2_.

For this problem, we set *θ*_2 _as 0.6*n *because it often occurs that the algorithm can find the desired attractor if *θ*_2 _≪ 0.6*n*, and that it hardly finds the desired attractor if *θ*_2 _≫ 0.6*n*.

## Results

### Results on simultaneous attractor control

The proposed method for SIMULTANEOUS ATTRACTOR CONTROL was applied to randomly generated BNs. The following data is according to 10 randomly generated pairs of BNs with *K *= 2, where for each pair of BNs, the BNs have the same nodes but different Boolean functions.

1. time: average CPU time (seconds) per pair of BNs,

2. #pos#rep: the number of pairs of BNs where the desired attractors can be found, and the average number of repeats for each such pair of BNs (recall that it is necessary for SAC (Simultaneous Attractor Control) to solve ILP instances repeatedly),

3. #neg#rep: the number of pairs of BNs for which the desired attractor does not exist, and the average number of repeats for each such pair of BNs.

We set *α_i _*= 1 and *γ_i _*= 1 for all *i *∈ {1, 2, ⋯, *n*}, and we set the maximum number of repeats to be 20. It is possible that this procedure may not decide whether or not the desired attractors exist within 20 repeats in some cases. The table shows that the number of repeats is small even though the number of nodes is large. The reason the CPU time for (140, 14) is greater than that for (160, 16) is that the latter required fewer repeats.

### Results on attractor control for normal cells under damaging cancer cell substantially

For this problem, we first guarantee that this control damages the cancer cell substantially and add the additional constraint (1). It is possible that no singleton attractor exists for the normal cell under the condition that the control set guarantees significant damage to the cancer cells. As shown in Table 3, our proposed method is useful also for this problem.

**Table 3:** Results on Attractor Control for the Normal Cells Given that the Control Damage the Cancer Cells Significantly

*n/m*	100/10	120/12	140/14	160/16
time(sec)	3.24	4.47	6.84	8.94
#pos/#rep	7/5	6/2.33	4/3.75	5/5.8
#neg/#rep	1/2	1/2	3/2	3/3

**Table 4:** Results on Attractor Control for the Cancer Cells Given that the control has limited Damage to the Normal Cells

*n/m*	100/10	120/12	140/14	160/16
time(sec)	3.12	4.05	5.89	8.21
#pos/#rep	8/2.88	5/2.6	4/1.5	4/3.5
#neg/#rep	2/2	4/1	4/1	4/1.5

### Results on attractor control for cancer cells under keeping normal cells undamaged

The results also suggest that our proposed approach is efficient and effective for solving the problem of attractor control of the cancer cell with no damage to the normal cell guaranteed.

**Figure 2:**
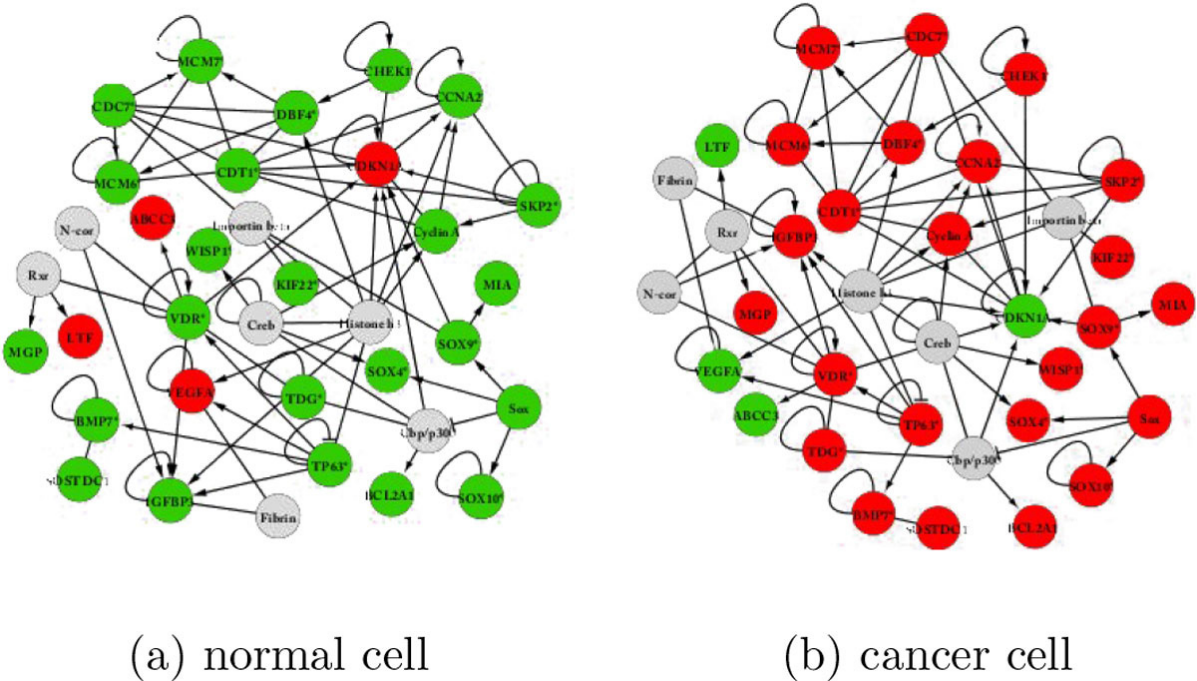
**Normal cell vs cancer cell**.

In order to verify our proposed method further, we applied ILP to some real networks (see Figure 5B) in [16]. There are two types of mice in that figure. The left figure is for BALB/c, which is more sensitive to radiation and tends to become cancer, while the right one is for C57BL/6, which is more resistant to radiation and tends not to become cancer. However, the nodes for these two networks are different and the Boolean functions are not given. Thus, we consider utilizing the right figure, which corresponds to the normal cell, and further simulating a cancer cell based on the normal cell (right figure) to guarantee that both networks have the same nodes (see Figure 2(b)). Furthermore, the red and green nodes for the normal cell represent 1 and 0, respectively. We assign most of the OR nodes to the cancer cell, and we see that the majority of nodes in the singleton attractor of the cancer cell are 1. Similarly, we assign most of the AND nodes to the normal cell, and we see that the majority of nodes in the singleton attractor of the normal cell are 0. This may mean that this is a subnetwork activated by cancer. Thus we have obtained the singleton attractor for the cancer cell and normal cell. Since our objective is to transform the state of the cancer cell into that of the normal cell, we try to find a control such that most of the 1 nodes in the cancer cell are converted into 0 nodes. Then we define a score function for both BNs. Specifically, we assume *γ_i _*= 1 and *α_i _*= 1 for all *i*, and we set *n *= 36, *m *= 4 and *θ *= 1.2·*n*. We can see that most of the states of the singleton attractor for cancer cells are changed into 0, indicating they are the desired attractors for the cancer cell under this control. Moreover, the CPU time is 0.03 (sec), which suggests that the proposed method might work efficiently for real medium-size networks.

## Discussions

In this paper, we formulated three novel problems, simultaneous attractor control for multiple networks, attractor control for normal cells with guaranteed damage to cancer cells, and attractor control for cancer cells guaranteed not to damage normal cells, and we applied an ILP-based approach for solving these problems in a unified manner. We further investigated the attractor control problem for multiple BNs and validated our proposed method for realistic networks. Though attractor control problems are Σ*p*-hard, the experimental results have shown the efficiency of our proposed method. Furthermore, this method was seen to be useful for solving medium-size instances of these problems. The method we proposed might not be the fastest, but it is easy and simple to implement and, furthermore, it has rooms for modifications and extensions. In particular, the use of non-linear costs for the scoring functions is of interest for future work.

## Competing interests

The authors declare that they have no competing interests.

## Authors' contributions

The basic idea of this research was proposed by TA after some discussion with WC. YQ and TT analyzed the theoretical details. All computer experiments were conducted by YQ. YQ wrote most parts of the manuscript, and TT edited it. TA and WC supervised the work. All authors read and approved the final manuscript.

## Declarations

Publication of this article was funded by University Grants of Kyoto University, Japan.

This article has been published as part of *BMC Systems Biology *Volume 8 Supplement 1, 2014: Selected articles from the Twelfth Asia Pacific Bioinformatics Conference (APBC 2014): Systems Biology. The full contents of the supplement are available online at http://www.biomedcentral.com/bmcsystbiol/supplements/8/S1.
